# Differential Roles of Cystathionine Gamma-Lyase and Mercaptopyruvate Sulfurtransferase in Hapten-Induced Colitis and Contact Dermatitis in Mice

**DOI:** 10.3390/ijms24032659

**Published:** 2023-01-31

**Authors:** Noriyuki Akahoshi, Ryoka Hasegawa, Shingo Yamamoto, Rintaro Takemoto, Toshiki Yoshizawa, Waka Kamichatani, Isao Ishii

**Affiliations:** Department of Health Chemistry, Showa Pharmaceutical University, Machida, Tokyo 194-8543, Japan

**Keywords:** contact dermatitis, cystathionine γ-lyase, cytokine, hydrogen sulfide, inflammatory bowel disease, mercaptopyruvate sulfurtransferase, systemic immunity, Th1/Th2 balance, trinitrochlorobenzene, ulcerative colitis

## Abstract

Hydrogen sulfide (H_2_S) has been shown to act as both anti-inflammatory and pro-inflammatory mediators. Application of H_2_S donors generally protects against inflammation; however, experimental results using mice lacking endogenous H_2_S-producing enzymes, such as cystathionine γ-lyase (CTH) and mercaptopyruvate sulfurtransferase (MPST), are often contradictory. We herein examined two types of model hapten-induced inflammation models, colitis (an inflammatory bowel disease model of mucosal immunity) and contact dermatitis (a type IV allergic model of systemic immunity), in CTH-deficient (*Cth*^–/–^) and MPST-deficient (*Mpst*^–/–^) mice. Both mice exhibited no significant alteration from wild-type mice in trinitrobenzene sulfonic acid (Th1-type hapten)-induced colitis (a Crohn’s disease model) and oxazolone (Th1/Th2 mix-type; Th2 dominant)-induced colitis (an ulcerative colitis model). However, *Cth*^–/–^ (not *Mpst*^–/–^) mice displayed more exacerbated phenotypes in trinitrochlorobenzene (TNCB; Th1-type)-induced contact dermatitis, but not oxazolone, at the delayed phase (24 h post-administration) of inflammation. CTH mRNA expression was upregulated in the TNCB-treated ears of both wild-type and *Mpst*^–/–^ mice. Although mRNA expression of pro-inflammatory cytokines (IL-1β and IL-6) was upregulated in both early (2 h) and delayed phases of TNCB-triggered dermatitis in all genotypes, that of Th2 (IL-4) and Treg cytokines (IL-10) was upregulated only in *Cth*^–/–^ mice, when that of Th1 cytokines (IFNγ and IL-2) was upregulated in wild-type and *Mpst*^–/–^ mice at the delayed phase. These results suggest that (upregulated) CTH or H_2_S produced by it helps maintain Th1/Th2 balance to protect against contact dermatitis.

## 1. Introduction

Inflammatory bowel disease (IBD) is a serious chronic condition of the colon and small intestine associated with severe pain, bleeding, and diarrhea, comprising two common forms: Crohn’s disease (CD) and ulcerative colitis (UC) [1]. CD is characterized by patchy lesions, potentially scattered in any area of the gastrointestinal tract, and transmural inflammation involving all bowel wall layers that leads to fibrosis, stricture, and fistula [2]. UC is characterized by mucosal inflammation from the rectum and continuously extends to the proximal colon [3]. Although both environmental and genetic factors, as well as several inflammatory mediators (cytokines and chemokines), have been implicated in their pathogenesis [2,4,5], the underlying molecular mechanisms are not yet fully elucidated.

Several clinical studies have suggested that colonic luminal hydrogen sulfide (H_2_S), with levels regulated by sulfate-reducing bacteria or colonic enzymes, such as H_2_S-producing enzymes, cystathionine γ-lyase (CTH; also known as CSE), and mercaptopyruvate sulfurtransferase (MPST; 3-MST), and H_2_S-degrating thiosulfate sulfurtransferase (TST; rhodanese) could be implicated in the IBD pathogenesis, especially UC [6,7]. Several experimental findings show that H_2_S (or its effects via persulfidation/polysulfidation [8,9]) plays important (patho)physiological roles in the neuronal, cardiovascular, and endocrine systems, as well as inflammation; importantly, exogenous/endogenous H_2_S could act as both anti-inflammatory and pro-inflammatory mediators, like a coin with two sides, perhaps depending on its doses or circumstances [10,11,12]. Therefore, this study examined the regulatory roles of CTH and MPST in experimental colitis induced by different model haptens, trinitrobenzene sulfonic acid (TNBS) and oxazolone, as mucosal immunity models [13], and experimental contact dermatitis induced by trinitrochlorobenzene (TNCB) and oxazolone, as systemic immunity models, using the previously established CTH-deficient (*Cth*^–/–^) and MPST-deficient (*Mpst*^–/–^) mice [14,15].

Contact dermatitis is the most common inflammatory condition caused by the exposure to exogenous substances that elicit skin and/or mucous membrane inflammation, and is estimated to affect 15–20% of the adult general population throughout a lifetime [16,17]. Although the therapeutic efficacy of H_2_S is expected for skin diseases [18], allergic contact dermatitis from diallyl trisulfide, a fast H_2_S donor, has been reported [19]. We herein found that *Cth*^–/–^ and *Mpst*^–/–^ mice and wild-type (WT) mice have similar responses to both TNBS- and oxazolone-induced colitis; however, *Cth*^–/–^ mice displayed the most severe phenotype in TNCB (not oxazolone)-induced contact dermatitis.

## 2. Results

### 2.1. Normal Responses of Cth^–/–^ and Mpst^–/–^ Mice in TNBS or Oxazolone-Induced Colitis

**Figure 1:**
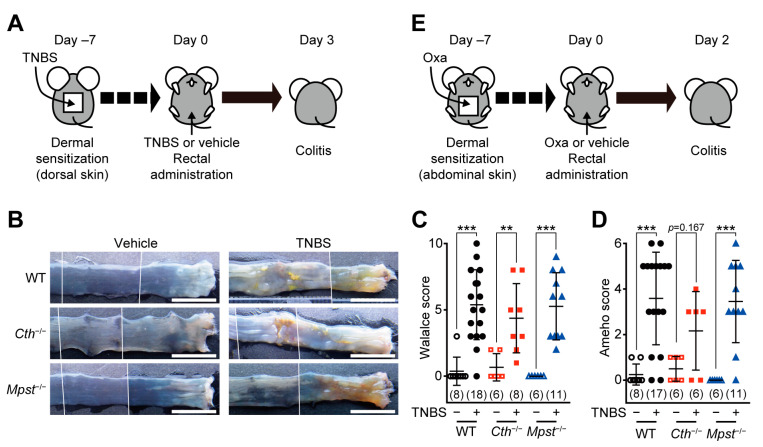

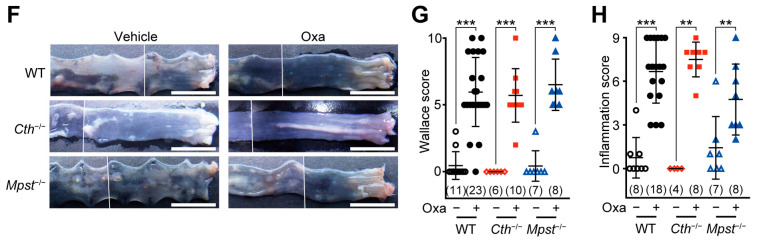
Two types of hapten-induced mouse IBD models: trinitrobenzene sulfonic acid (TNBS)-induced Crohn’s disease-like model (**A**–**D**) and oxazolone (Oxa)-induced ulcerative colitis-like model (**E**–**H**). (**A**,**E**) Experimental Design. Adult male wild-type (WT), CTH-deficient (*Cth*^–/–^), and MPST-deficient (*Mpst*^–/–^) mice were sensitized on the (dorsal or abdominal) skin and then (7 days later) challenged per rectum with those haptens. (**B**–**D**) Typical pictures of colonic lumens (**B**), Wallace scores (**C**), and Ameho scores (**D**), obtained with histological examination of colonic sections (Supplementary Figure S1) at day 3 after the TNBS challenge. (**F**–**H**) Typical pictures of colonic lumens (**F**), Wallace scores (**G**), and inflammation scores (**H**), obtained with histological examination of colonic sections (Supplementary Figure S2) at day 2 after the Oxa challenge. Data are the mean ± standard derivation (SD) with sample numbers in parentheses. Differences between non-sensitized (vehicle alone) and pre-sensitized (with hapten) are significant in a Mann–Whitney U test at ** *p* < 0.01 and *** *p* < 0.001 (**C**,**D**,**G**,**H**). Bars indicate 1 cm (**B**,**F**).

Experimental colitis was induced by the initial topical sensitization, followed by rectal administration of TNBS (as a Th1/Th17-associated CD model [20]; Figure 1A) or oxazolone (as a Th1/Th2-associated UC model [20]; Figure 1E) in adult male WT, *Cth*^–/–^, and *Mpst*^–/–^ mice. All TNBS-treated groups displayed granulomatous ulcers in the colonic lumens (Figure 1B), which resemble CD phenotypes [2], and thus, had higher Wallace scores than the respective vehicle-treated groups (Figure 1C), similar in all genotypes. Histological analyses of hematoxylin/eosin (HE)-stained sections revealed transmural inflammation that extends into the muscular layers of the colon (Supplementary Figure S1), similar to higher Ameho scores (Figure 1D), in all TNBS-treated groups. Challenges with the oxazolone challenge caused more superficial ulcers (like UC patients [3]) that continue from the anuses to the colonic lumens (Figure 1F) in all three genotypes. Oxazolone treatment also increased Wallace scores (Figure 1G) and induced extensive erosive inflammation (Supplementary Figure S2), resulting in increased inflammation scores to the equivalent levels in the three genotypes (Figure 1H).

### 2.2. Exacerbated TNCB (not Oxazolone)-Induced Contact Dermatitis in Cth^–/–^ Mice

**Figure 2:**
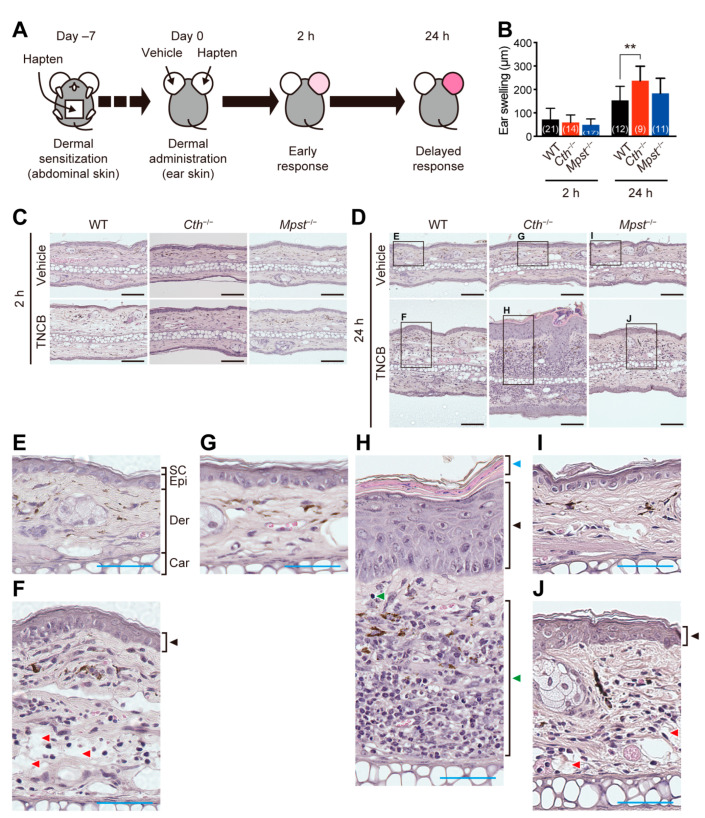
Trinitrochlorobenzene (TNCB)-induced contact dermatitis. (**A**) Experimental design. Adult male wild-type (WT), CTH-deficient (*Cth*^–/–^), and MPST-deficient (*Mpst*^–/–^) mice were sensitized on the skin and then (7 days later) challenged with TNCB on single ear (another ear with the vehicle) to examine Th1-type allergic responses. (**B**–**J**) Increased thickness of the ear (ear swelling = thickness difference between the TNCB-treated right ear and the vehicle-treated left ear) at 2 h and 24 h after the challenge (**B**) and their typical section images of HE-stained sagittal sections (**C**,**D**) with magnified images (**E**–**J**). Data are the mean ± SD (sample numbers). Differences are significant between the groups at each point by a one-way analysis of variance (ANOVA) with Tukey’s multiple comparison test; ** *p* < 0.01 (**B**). Black bars indicate 100 µm (**C**,**D**), and blue bars indicate 50 µm (**E**–**J**). SC, stratum corneum; Epi, epidermis; Der, dermis; and Car, cartilage (**E**). Black arrowheads indicate TNCB-induced acanthosis (**F**,**H**,**J**); blue and green arrowheads indicate parakeratosis and immune cell infiltration, respectively (**H**); and red arrowheads indicate spongiosis (**F**,**I**).

Experimental contact dermatitis was induced by the initial sensitization of TNCB or oxazolone on the abdominal skin, followed by the elicitation with the same hapten on one of the ears and the vehicle on another ear (Figure 2A), as models, to evaluate Th1- and Th1/Th2 mix-type allergic responses, respectively [21]. The elicitation response can be divided into two phases: the early phase at 2 h and the delayed phase at 24 h, after the challenge [21]. Allergic responses caused by TNCB-induced acute ear thickening after 2 h, followed by the delayed responses with additional thickening after 24 h in all three genotypes, although *Cth*^–/–^ mice exhibited more severe thickening than WT and *Mpst*^–/–^ mice at the delayed phase (Figure 2B–D). Closer examination of HE-stained sections revealed that TNCB induced acanthosis (epidermal hyperplasia; black arrowheads in Figure 2F,J) and spongiosis (red arrowheads in Figure 2F,J) in both WT and *Mpst*^–/–^ mice, but induced immune cell infiltration only in *Cth*^–/–^ mice (green arrowheads in Figure 2H). However, acanthosis and immune cell infiltration (especially lymphocyte and granule cell infiltration into the dermis) were more pronounced, whereas spongiosis was rather inconspicuous in *Cth*^−/−^ mice (Figure 2G,H).

**Figure 3:**
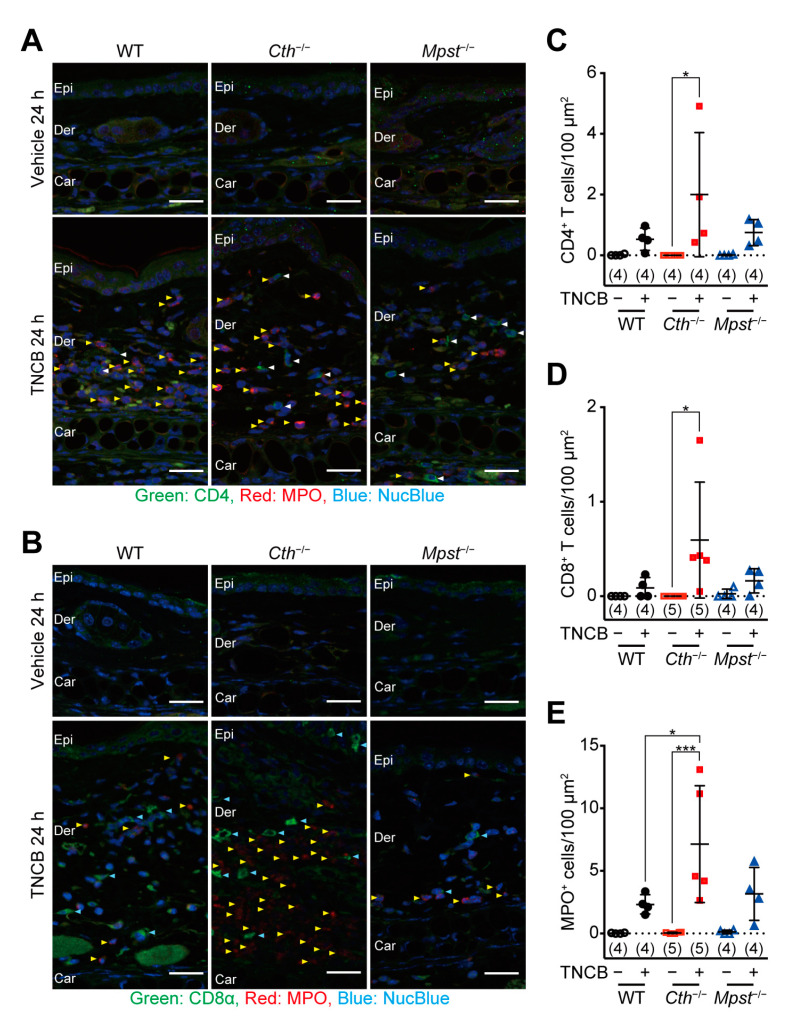
Immunohistochemical analysis of ear sagittal sections at 24 h after the trinitrochlorobenzene (TNCB) challenge. (**A**) Coimmunostaining with anti-CD4 antibody (green), anti-myeloperoxidase (MPO) antibody (red), and NucBlue (to stain nuclei, blue). White and yellow arrowheads indicate CD4^+^ cells and MPO^+^ cells, respectively. (**B**) Coimmunostaining with anti-CD8α antibody (green), anti-MPO antibody (red), and NucBlue (blue). Light blue and yellow arrowheads represent CD8^+^ and MPO^+^ cells, respectively. SC, stratum corneum; Epi, epidermis; Der, dermis; and Car, cartilage in (**A**,**B**). (**C**–**E**) Distribution density of CD4^+^ (**C**), CD8^+^ (**D**), and MPO^+^ (**D**) cells per 100 µm^2^. Data are the mean ± SD (sample numbers). Differences between groups in each time point are significant by a one-way ANOVA with Tukey’s multiple comparison test at * *p* < 0.05 and *** *p* < 0.001 (**C**–**E**). White bars indicate 25 µm (**A**,**B**).

**Figure 4:**
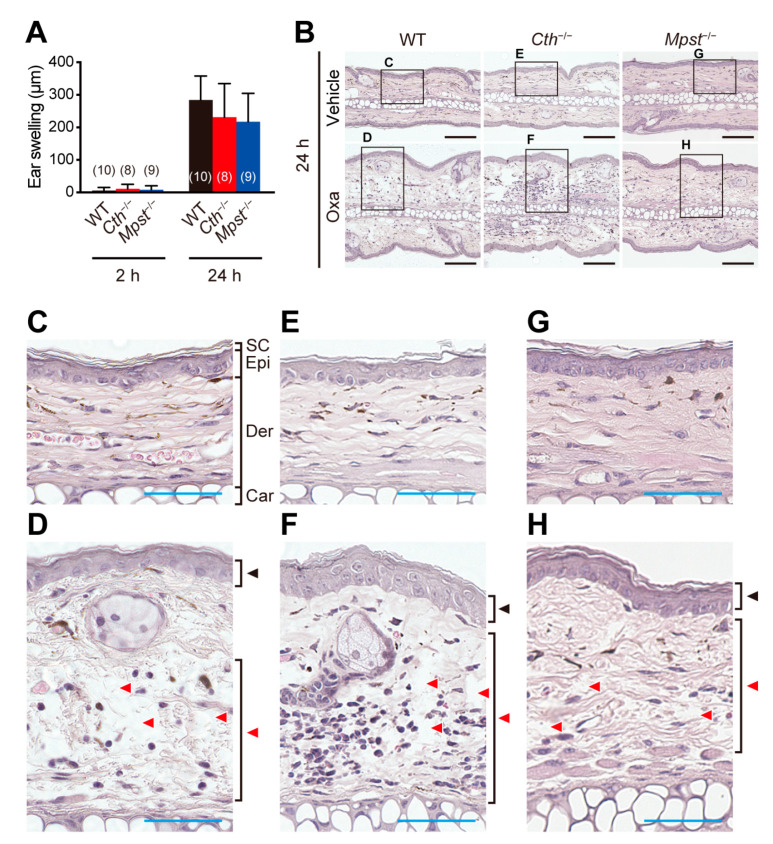
Oxazolone-induced contact dermatitis. Adult male wild-type (WT), CTH-deficient (*Cth*^–/–^), and MPST-deficient (*Mpst*^–/–^) mice were sensitized on the skin and then (7 days later) challenged with oxazolone (Oxa) on a single ear (another ear with the vehicle) to examine Th1/Th2-type allergic responses. (**A**) Increased ear thickness (the difference between oxazolone-treated right ear and vehicle-treated left ear) at 2 h and 24 h after oxazolone challenge. Data are the mean ± SD (sample numbers). (**B**–**H**) Typical section images of HE-stained sagittal sections of ears at 24 h after oxazolone challenge (**B**) with their magnified images (**C**–**H**). SC, stratum corneum; Epi, epidermis; Der, dermis; and Car, cartilage in (**A**,**B**). Black arrowheads indicate oxazolone-induced acanthosis, whereas red arrowheads indicate spongiosis (**D**,**F**,**H**). Bars indicate 100 µm (black, **B**) or 50 µm (blue, **C**–**H**).

Immunostaining of ear sections revealed the most massive infiltration of CD4-positive T cells (Figure 3A,C), CD8-positive T cells (Figure 3B,D), and myeloperoxidase (MPO)-positive neutrophils (Figure 3A,B,E) in *Cth*^−/−^ mice, compared to WT and *Mpst*^–/–^ mice. Acute allergic responses (within 2 h) were not observed with oxazolone treatment, although the levels of their delayed responses (at 24 h) were comparable to those with TNCB and equivalent among the three genotypes (Figure 4A). HE staining revealed similar levels of acanthosis, dermal spongiosis, and immune cell infiltration in the three genotypes (Figure 4B–H).

### 2.3. CTH mRNA Induction in the Delayed Phase of TNCB-Induced Contact Dermatitis

**Figure 5:**
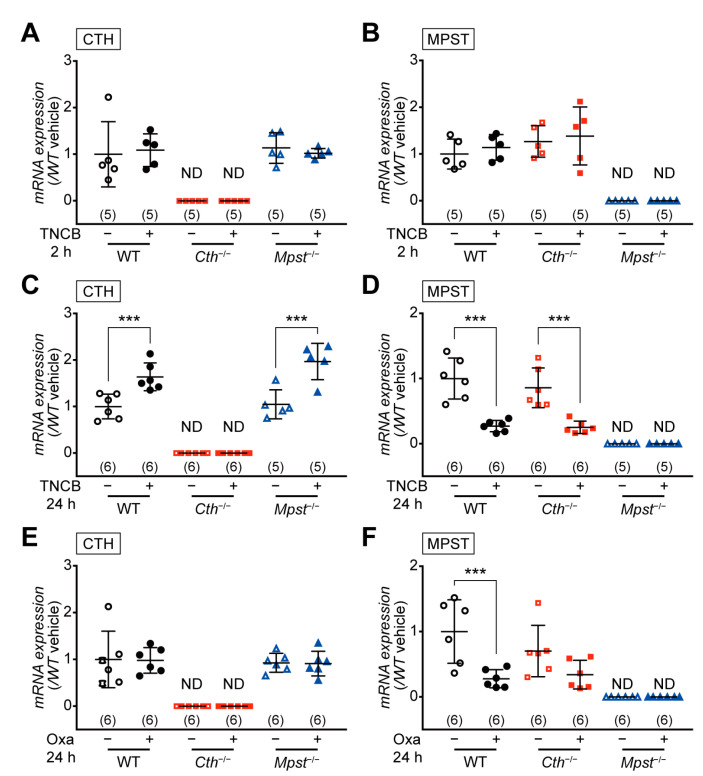
Expression levels of CTH and MPST mRNAs in the ear during hapten-induced contact dermatitis. The total RNA was extracted from each ear at 2 h (**A**,**B**) or 24 h (**C**–**F**) after trinitrochlorobenzene (TNCB; **A**–**D**) or oxazolone (Oxa; **E**,**F**) challenge. CTH (**A**,**C**,**E**) and MPST (**B**,**D**,**F**) mRNA levels were normalized by the housekeeping HPRT1 mRNA levels, and the relative expression versus vehicle-treated wild-type samples were calculated. Data are the mean ± SD (sample numbers) in parentheses. Differences are significant by a one-way ANOVA with Tukey’s multiple comparison test at *** *p* < 0.001. ND, not detectable.

Quantitative reverse transcription-polymerase chain reaction (RT-PCR) analyses revealed no level changes in both CTH and MPST mRNA in the TNCB- and vehicle-treated ears of WT, *Mpst*^–/–^ (without *Mpst*), and *Cth*^–/–^ mice (without *Cth*) at the early phase (2 h) (Figure 5A,B). However, at the delayed phase (24 h), CTH mRNA was upregulated in the TNCB-treated ears of WT and *Mpst*^–/–^ mice, whereas MPST mRNA was downregulated in the TNCB-treated ears of WT and *Cth*^–/–^ mice (Figure 5C,D), suggesting protective roles of CTH against the delayed phase of TNCB-induced contact dermatitis. MPST downregulation was also found in the delayed phase (not the early phase) of oxazolone-treated ears of WT and *Cth*^–/–^ mice (but not significant in *Cth*^–/–^) (Figure 5E,F); therefore, MPST mRNA downregulation may not be implicated in the disease condition.

### 2.4. Marked Induction of Pro-inflammatory/Th2 Cytokine mRNA in the Delayed Phase of TNCB-Induced Contact Dermatitis in Cth^–/–^ Mice

**Figure 6:**
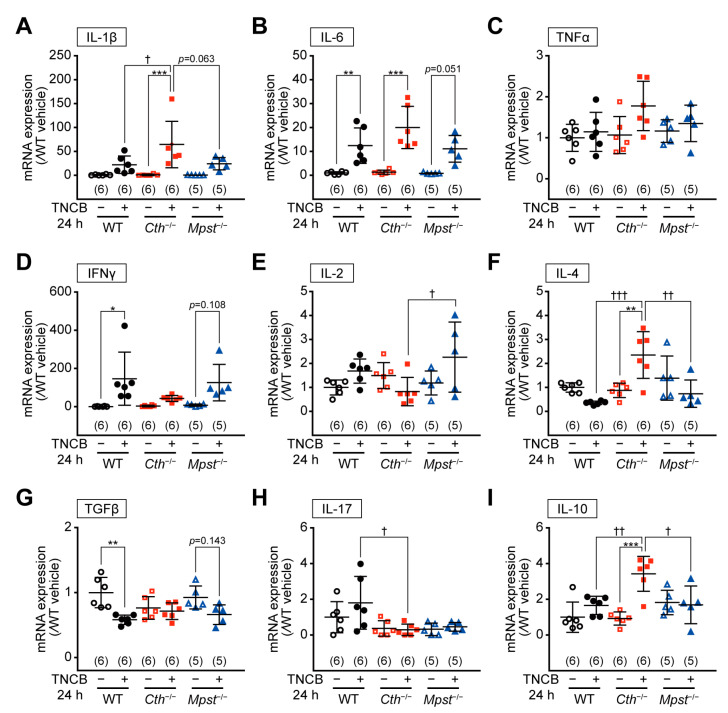
Expression levels of various cytokine mRNAs in the ear during trinitrochlorobenzene (TNCB)-induced contact dermatitis at 24 h after the challenge. IL-1β (**A**), IL-6 (**B**), TNFα (**C**), IFNγ (**D**), IL-2 (**E**), IL-4 (**F**), TGFβ (**G**), IL-17 (**H**), and IL-10 (**I**) mRNA levels were normalized by the housekeeping HPRT1 mRNA levels, and the relative expression versus vehicle-treated wild-type samples were calculated. Data are the mean ± SD with sample numbers in parentheses. Differences are significant by a one-way ANOVA with Tukey’s multiple comparison test at * *p* < 0.05, ** *p* < 0.01, and *** *p* < 0.001 versus vehicle-treated samples of each genotype; ^†^ *p* < 0.05, ^††^ *p* < 0.01, and ^†††^ *p* < 0.001 between genotypes.

**Figure 7:**
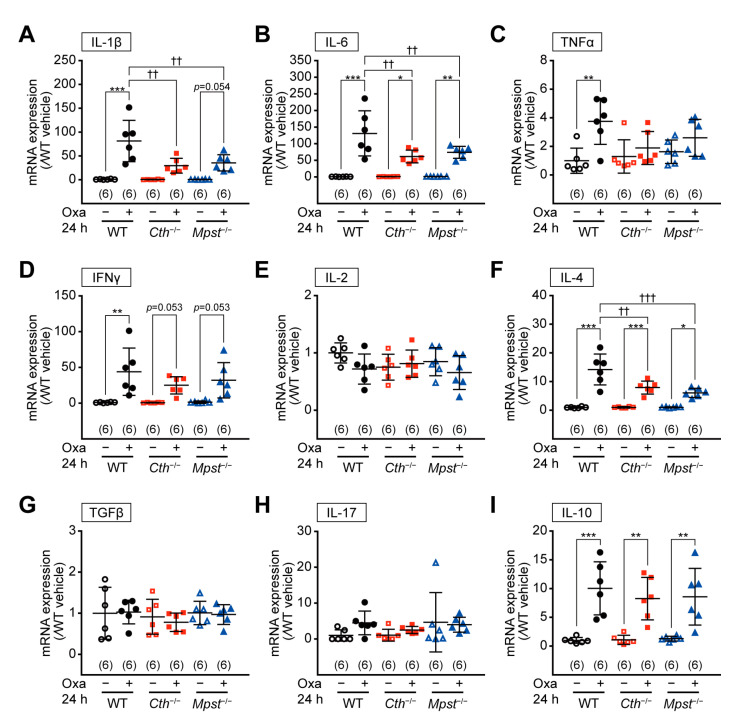
Expression levels of various cytokine mRNAs in the ear during oxazolone (Oxa)-induced contact dermatitis at 24 h after the challenge. IL-1β (**A**), IL-6 (**B**), TNFα (**C**), IFNγ (**D**), IL-2 (**E**), IL-4 (**F**), TGFβ (**G**), IL-17 (**H**), and IL-10 (**I**) mRNA levels were normalized by the housekeeping HPRT1 mRNA levels, and the relative expression versus vehicle-treated wild-type samples were calculated. Data are the mean ± SD with sample numbers in parentheses. Differences are significant by a one-way ANOVA with Tukey’s multiple comparison test at * *p* < 0.05, ** *p* < 0.01, and *** *p* < 0.001 versus vehicle-treated samples of each genotypes; ^††^ *p* < 0.01 and ^†††^ *p* < 0.001 between genotypes.

RT-PCR analyses revealed mRNA upregulation of pro-inflammatory cytokines, interleukin 1 beta (IL-1β)*,* interleukin 6 (IL-6), and TNFα (but not significant), without apparent mRNA alterations in cytokines that regulate T-cell differentiation, such as interferon gamma (IFNγ), interleukin 2 (IL-2), interleukin 4 (IL-4), tumor growth factor beta (TGFβ), interleukin 17 (IL-17), and interleukin 10 (IL-10), in the early phase of TNCB- and vehicle-treated ears of all genotypes (Supplementary Figure S3; except for slight IL-4 mRNA upregulation in *Cth*^–/–^ mice). In contrast, at the delayed phase, mRNA upregulation of IL-1β and IL-6 was the most pronounced and that of Th2 cytokines (IL-4 and IL-10) was only observed in the TNCB-treated ears of *Cth*^–/–^ mice (Figure 6A–I). In the TNCB-treated ears of WT and *Mpst*^−/−^ mice, Th1 cytokines (IFNγ and IL-2) expression was somewhat upregulated (Figure 6D,E, respectively), whereas that of Th2 cytokine IL-4 was downregulated (Figure 6F), supporting TNCB as a Th1-type inflammation-inducing hapten. Conversely, such changes were not apparent in *Cth*^–/–^ mice (Figure 6D–F). Both TGFβ and IL-17 mRNA were not upregulated in TNCB-treated ears of all genotypes (Figure 6G,H, respectively), and therefore, Th17 responses may not be involved. RT-PCR analyses also revealed mRNA upregulation of pro-inflammatory cytokines (IL-1β and IL-6) and some Th1 or Th2 cytokines (IFNγ, IL-4, and IL-10), but not that of IL-2, TGFβ, and IL-17, at the delayed phase of oxazolone-induced contact dermatitis, similar within the three genotypes (Figure 7A–I).

## 3. Discussion

The first quarter of a century has passed since the first identification of H_2_S as the signaling gaseous molecule; to date, >60,000 publications (in PubMed) have reported its versatile roles in regulating neural, cardiovascular, and endocrine systems, as well as inflammation (reviewed in [10,22]). Notably, H_2_S could function as either an anti-inflammatory or pro-inflammatory mediator [10,11,12]. H_2_S is apt to show anti-inflammatory effects at relatively low or physiological concentrations, whereas it exerts pro-inflammatory effects when the tissues/cells are exposed to high concentrations or under particular disease conditions, such as some types of inflammation [10,11,12]. Although applications of the so-called H_2_S donors generally exert anti-inflammatory, cytoprotective, and antioxidant effects in the cells and animals [10], they often result in enhanced inflammation [23]. Indeed, our *Cth*^–/–^ mice displayed the systemic resistance against caerulein-induced acute pancreatitis [24], cecal-ligation and puncture-induced sepsis [25], and acute renal ischemia/reperfusion injury and its associated inflammatory responses [26], suggesting the pro-inflammatory roles of endogenous H_2_S produced by CTH at those inflammation loci.

As for experimental colitis, H_2_S donor application has been shown to alleviate DSS-induced colitis in mice [27,28] and rats [29], and TNBS-induced colitis in mice [30] and rats [31,32]. DSS-induced colitis is one of the most commonly used IDB models to evaluate drug candidates because this model can be easily induced by administering DSS by drinking water, although this model is fundamentally different from hapten-induced colitis [13]. Treatment with a nonspecific CTH inhibitor propargylglycine exacerbated DSS-induced colitis in mice [33], and mRNA expression of three H_2_S-producing enzymes (CTH, MPST, and cystathionine β-synthase [CBS]) and H_2_S production was upregulated in the colon mucosa in the experimental colitis of mice/rats [29,33,34,35]. *Cth*^–/–^ mice of different origins were more susceptible to DSS-induced colitis [28] and dinitrobenzene sulfonic acid (DNBS [TNBS analog]; single intracolonic administration)-induced colitis [36], whereas *Mpst*^–/–^ mice of a different origin [37] were more vulnerable to both TNBS- and DSS-induced colitis [35]. In this study, both *Cth*^–/–^ and *Mpst*^–/–^ mice (on the same C57BL/6J background) did not show significantly altered responses to TNBS and oxazolone (Figure 1A–F). Discrepancies between these studies could be attributable to the differences of experimental procedures, stimulants (DSS, DNBS, or TNBS), administration routes (oral or per rectum), a single administration or the challenge after sensitization, and fasted or not before the challenge.

On the other hand, the aberrant H_2_S metabolism has been involved in the pathogenesis of various skin diseases, including vascular disorders, pigmentation disorders, melanoma, ulcers, and psoriasis [18]. Psoriasis, which is characterized by hyper-proliferative keratinocytes and auto-reactive immune cells, was associated with low serum levels of H_2_S and high IL-6, IL-8, and TNFα [38]. H_2_S donor application to mice alleviated chemically induced itch [39], promoted skin wound healing via oxidative stress inhibition and vascular endothelial growth factor enhancement [40], and improved diabetic wound healing by inhibiting NETosis and NETs release [41]. In contrast, the mean disulfide levels were found to be significantly higher in the rosacea patients than in the control group [42], and the serum levels of H_2_S were significantly higher in atopic dermatitis patients compared to healthy controls [43]. Based on those somewhat contradictory findings, hapten-induced contact dermatitis (also referred to as contact hypersensitivity) was first investigated in *Cth*^–/–^ and *Mpst*^–/–^ mice as another inflammation model of the systemic immunity. Experimental contact dermatitis consists of the early phase dominated by complement and innate immune cells and the delayed phase governed by adaptive immune cells, such as CD4^+^ T cells (Th1 and Th2 cells), CD8^+^ T cells (Tc1 cells), and B cells, as a typical type IV allergic response [44,45].

Our *Cth*^−/−^ mice (but not *Mpst*^–/–^ mice) displayed higher sensitivity against TNCB at the delayed inflammation phase, as revealed by ear thickening (Figure 2B,D,H), infiltration of CD4^+^ and CD8^+^ T cells and MPO^+^ neutrophils (Figure 3A–E), and mRNA induction of pro-inflammatory cytokines (IL-1β and IL-6) and Th2-type cytokines (IL-4 and IL-10) (Figure 6A,B,F,I). Conversely, both *Cth*^–/–^ and *Mpst*^–/–^ mice did not display marked alteration from WT mice in oxazolone-induced contact dermatitis at the delayed phase (Figure 4A–H) and mRNA upregulation of cytokines in all phases (Figure 7A–I). Notably, CTH mRNA was upregulated, whereas MPST mRNA was downregulated at the delayed phase of TNCB-treated ears (Figure 5C,D), when CTH mRNA upregulation was not observed in the early phase of TNCB-treated ears (Figure 5A) and the delayed phase of oxazolone-induced ears (Figure 5E), and MPST mRNA downregulation, was evident in the delayed phase of oxazolone-induced ears (Figure 5F).

These results suggest that CTH in the ear protected against the delayed phase of TNCB-induced inflammation by suppressing Th1-to-Th2 shift, whereas CTH deletion in mice exacerbated the inflammation by Th1/Th2 imbalance. Because acute ear canal responses to passive cutaneous anaphylaxis were equivalent between WT, *Cth*^–/–^, and *Mpst*^–/–^ mice in our previous study [15], CTH or H_2_S might play more roles in the antigen-specific delayed phase of inflammation, rather than the antigen nonspecific early phase that mainly involves innate immune cells [46]. Pro-inflammatory M1 macrophages are activated by Th1 cytokines, whereas anti-inflammatory M2 macrophages are activated by Th2 cytokines. H_2_S produced by CBS has been shown to suppress the polarization of mouse primary microglia (macrophages in the brain) toward M1 phenotypes [47]. H_2_S regulated the expression of methylcytosine dioxygenases Tet1/Tet2 to promote regulatory T-cell differentiation in immune cells, and H_2_S deficiency led to systemic autoimmune disease [48]. Therefore, H_2_S produced by CTH could help maintain the Th1/Th2 balance against challenges with the specific model hapten (TNCB) in this study.

Hyperhomocysteinemia (a highly elevated blood homocysteine level) should also be considered in *Cth*^–/–^ mice; total serum homocysteine levels are 83.6 ± 18.6 µM (*n* = 5) and 5.78 ± 1.22 µM (*n* = 10) in WT mice, and 5.90 ± 2.05 µM (*n* = 7) in *Mpst*^–/–^ mice [15]. Elevated blood homocysteine levels are an independent risk factor for cardiovascular diseases and the risk of hyperhomocysteinemia is significantly higher in patients with IBD [49]. Diet (high methionine)-induced hyperhomocysteinemia exacerbated DSS-induced colitis in rats [50], and homocysteine promoted CD4^+^ T cell differentiation (lamina propria lymphocytes in the colonic mucosa of Wistar rats) into Th17 cells [51]. As for the skin, patients with psoriasis have significantly higher serum homocysteine levels [52], although the relationship between homocysteinemia and contact dermatitis remains unknown. Therefore, the exacerbated phenotypes in TNCB-induced contact dermatitis may not be solely attributable to H_2_S deficiency in *Cth*^–/–^ mice.

In conclusion, we have found that CTH plays important roles in protecting against contact dermatitis, induced by the specific model hapten (TNCB but not oxazolone). In this study, endogenous H_2_S produced by CTH could act as a specific anti-inflammatory mediator. Our previous study demonstrated the altered serum amino acid profiles in *Cth*^–/–^ and *Mpst*^–/–^ mice [15], and further studies are warranted to explain the complex and often opposing roles of H_2_S in inflammatory responses.

## 4. Materials and Methods

### 4.1. Animals

C57BL/6J inbred strain (C57BL/6JJcl; CLEA Japan, Tokyo, Japan) were used as WT mice. *Cth*^+/−^ mice were generated by homologous recombination in embryonic stem cells and blastocyte injection, and backcrossed for 12 generations to C57BL/6JJcl [14]. *Mpst*^+/−^ mice were generated using CRISPR/Cas9 technology on fertilized C57BL/6JJcl embryos by Setsurotech Inc. (Tokushima, Japan) [15]. Heterologous mice were bred to obtain homozygous mice, and these mice (8–22-week-old males) were used for comparative analyses on the same C57BL/6JJcl background. Preoperatively, mice were housed in an air-conditioned room (23 ± 1 °C, 55 ± 5% humidity), kept in a 12 h dark/light cycle, and allowed free access to a CE-2 standard dry rodent diet (CLEA Japan) and water. Before the surgeries, mice were anaesthetized with isoflurane inhalation.

### 4.2. Hapten-Induced Colitis

Hapten-induced experimental colitis was established as IBD models in mice. Adult male mice (aged 7–21 weeks) were anesthetized with isoflurane and sensitized by the topical application of 150 µL of 1% (*v/v*) TNBS (Sigma-Aldrich, Burington, MA, USA; cat no. P2297) in the vehicle (acetone-olive oil, 4:1 [*v/v*]) to the shaved dorsal skin (1.5 × 1.5-cm^2^) between the shoulders. After 7 days, mice were weighed, anesthetized with isoflurane, and administered (~30 s) with 100 µL of 50% ethanol (vehicle) or 2.5% (*w/v*) TNBS in the vehicle, per rectum, using a 3.5-Fr. catheter system (Nippon Sherwood Medical Industries, Osaka, Japan). Mice were also sensitized by topical application of 150 µL of 3% (*w/v*) oxazolone (Sigma-Aldrich; cat no. 862207) in ethanol to the shaved ventral skin (2 × 2-cm square). After 7 days, they were weighed, anesthetized with isoflurane, and administered (~30 s) with 100 µL of 50% ethanol (vehicle) and or 1% (*w/v*) oxazolone in the vehicle, per rectum, using a 3.5-Fr. catheter system. After the administration, mice were kept upside-down for 30 s to prevent backflow. Mice were weighed daily and sacrificed at day 3 (for TNBS) or day 2 (for oxazolone), post-administration. The colons, including rectums, were quickly removed from the anesthetized mice, measured for their lengths, washed in saline, spread inside-out on a black paper, and examined for macroscopic lesions using the Wallace score, which scores on a scale from 0 to 10 based on the following criteria: hyperemia, bowel wall thickening, and the extent of ulceration and inflammation [53]. Then, the colon was fixed in either 10% formalin, dehydrated in an ascending ethanol series (from 75% to 100%), and then xylene, and embedded in paraffin. About 3-µm sections were cut, deparaffinized, and stained with HE. TNBS-induced colitis was examined using the Ameho score [54]. Oxazolone-induced colitis was examined using the inflammation score defined in this study to evaluate tissue anomaly with reference to Bendtsen et al. [55]. The anomaly in the mucosal surface (epithelial cell loss, depletion of mucin producing goblet cells, and/or reduction of tubular density) was defined as ulcer, and scores of 0–3 (0 = no change, 1 = mild, 2 = moderate, and 3 = general) were given by one person. Similarly, anomalies in the mucosal intrinsic layer (immune cell filtration) and submucosal layer (edema) were independently defined and scored 0–3 each. Thus, the maximum inflammation score is summed up to 9 (Figure 1H).

### 4.3. Hapten-Induced Contact Dermatitis

Hapten-induced contact dermatitis was examined for contact hypersensitivity. Adult male mice (7–21 weeks) were anesthetized with isoflurane and sensitized by the topical application of 100 µL of 7% (*v/v*) TNCB (Tokyo Chemical Industry, Tokyo, Japan) in acetone-olive oil (4:1, *v/v*) to the shaved abdomen (approximately 2 cm^2^). After 7 days, mice were challenged with 10 µL of 1% TNCB in acetone-olive oil (9:1, *v/v*) onto each side of the left ear, and the right ear was administered with the vehicle alone. The ear thickness was measured using a digital SMD-565J-L thickness gauge (Teclock, Nagano, Japan) before the induction (*T_0_*), and at 2 h (*T_1_*) and 12 h (*T_2_*) after the induction. The ear swelling (µm thickness) was calculated as (*T_1 or 2_*−*T*_0_ of the left ear)–(*T_1 or 2_*−*T*_0_ of the right ear). After 24 h measurement, the entire pinna was collected and weighed. The ear was cut lengthwise into two pieces; one was fixed in 10% formalin for hematoxylin-eosin staining of paraffin sections and one for mRNA expression assays. Mice were also sensitized with 100 µL of 3% oxazolone in ethanol and then (5 days later) challenged with 10 µL of 1% oxazolone in ethanol.

### 4.4. Immunohistochemistry

Paraffin sections were deparaffinized with xylene and ethanol, antigen-activated in Immunosaver (Fujifilm-Wako, Osaka, Japan) at 98 °C for 45 min, and then blocked with 5% normal donkey serum in phosphate-buffered saline-0.5% Tween 20. Sections were incubated with goat anti-myeloperoxidase (MPO) polyclonal antibody (1:500; Santa Cruz; cat no. sc-16129), rabbit anti-CD4 (BLR167J) monoclonal antibody (1:500; Bethyl Laboratories, Waltham, MA, USA; cat no. A700-167-T), or rabbit anti-CD8 alpha (BLR173J) monoclonal antibody (1:500; Bethyl Laboratories; cat no. A700-173-T) in Can Get Signal A (Toyobo, Osaka, Japan) as the primary antibody, and donkey anti-rabbit IgG conjugated Alexa 488 (1:500; Invitrogen, Waltham, MA, USA) and donkey anti-goat IgG conjugated Alexa 568 (1:500; Invitrogen) was used in the blocking solution as the secondary antibody. Sections were sealed with ProLong Glass Antifade Mountant with NucBlue Stain (ThermoFisher Scientific, Waltham, MA, USA) and observed by a BZ-9000 microscope (Keyence, Osaka, Japan) equipped with Nikon objectives.

### 4.5. Quantitative RT-PCR

The total RNA was isolated from the tissues using a TRI reagent (Molecular Research Center, Cincinnati, OH, USA), and cDNA was synthesized using a ReverTra Ace qPCR RT Master Mix with random hexamers (Toyobo). Quantitative real-time PCR assay was performed using THUNDERBIRD qPCR Mix (Toyobo) with a CFX Connect Real-Time PCR Detection System (Bio-Rad). Primers were designed using the Primer3 program (https://primer3.ut.ee, accessed on 1 January 2020) (Supplementary Table S1). The cycling conditions consisted of initial denaturation at 95 °C for 10 s, followed by 50 cycles of 95 °C for 5 s and 60 °C for 30 s.

### 4.6. Statistical Analyses

Data were expressed as mean ± SD with sample numbers in parentheses. Statistical comparison was performed by a Mann–Whitney U test or a one-way analysis of variance (ANOVA) with Tukey’s multiple comparison test using Prizm 5 software (GraphPad, San Diego, CA, USA). All *p-*values of <0.05 denoted a significant difference.

## Data Availability

Not applicable.

## Supplementary Materials

The following supporting information can be downloaded at: https://www.mdpi.com/article/10.3390/ijms24032659/s1.
