# Supplementary material for: Differential Roles of Cystathionine Gamma-Lyase and Mercaptopyruvate Sulfurtransferase in Hapten-Induced Colitis and Contact Dermatitis in Mice
Source: Int J Mol Sci. 2023 Jan 31;24(3):2659. doi: 10.3390/ijms24032659 (PMC9916491; doi:10.3390/ijms24032659)
Supplement: Supplementary file 1 [file ijms-24-02659-s001.zip › ijms-2165847-SI.pdf]

Supplementary Data

Differential Roles of Cystathionine Gamma-Lyase and Mercaptopyruvate Sulfurtransferase in Hapten-Induced Colitis and Contact Dermatitis in Mice

Noriyuki Akahoshi, Ryoka Hasegawa, Shingo Yamamoto, Rintaro Takemoto, Toshiki Yoshizawa, Waka Kamichatani and Isao Ishii*

Department of Health Chemistry, Showa Pharmaceutical University, Machida,
Tokyo 194-8543, Japan

* Correspondence: i-ishii@ac.shoyaku.ac.jp ; Tel.: +81-42-721-1563

Supplementary Table 1: Primer sets for quantitative RT-PCR.

Supplementary Figure 1: Hematoxylin/eosin-stained colon sagittal sections of TNBS-induced colitis in wild-type (WT), CTH-deficient (*Cth*$^{-/-}$), and MPST-deficient (*Mpst*$^{-/-}$) mice.

Supplementary Figure 2: Hematoxylin/eosin-stained colon sagittal sections of oxazolone-induced colitis in wild-type (WT), CTH-deficient (*Cth*$^{-/-}$), and MPST-deficient (*Mpst*$^{-/-}$) mice.

Supplementary Figure 3: Expression levels of various cytokine mRNAs in the ear during trinitrochlorobenzene (TNCB)-induced contact dermatitis at 2 h after the challenge. Levels of IL-1β (A), IL-6 (B), TNAα (C), IFNγ (D), IL-2 (E), IL-4 (F), TGFβ (G), IL-17 (H), and IL-10 (I) mRNA levels were normalized by the housekeeping HPRT1 mRNA levels and the relative expression *versus* vehicle-treated wild-type samples were calculated. Data are the mean ± SD with sample numbers in parentheses. Differences are significant by a one-way ANOVA with Tukey's multiple comparison test at *p<0.05, **p<0.01, and ***p<0.001 *versus* vehicle-treated samples of each genotype.

Supplementary Table 1 Primer sets for quantitative RT-PCR

Gene		Sequence (5'-3')	Amplicon size (base pair)
Cth	Forward	5'-TGGTGCTGCCCATTTCGTTG-3'	265
	Reverse	5'-GCCACCCTCCTGAAGTACCT-3'	
Hprt1	Forward	5'-ATTGTGGCCCTCTGTGTGCT-3'	165
	Reverse	5'-AACTTTTATGTCCCCCGTTGACT-3'	
Ifng	Forward	5'-TGGCTTTGCAGCTCTTCCTC-3'	157
	Reverse	5'-TCCTTTTGCCAGTTCCTCCA-3'	
Il1β	Forward	5'-AGCACCTTCTTTTCCTTCATCTTG-3'	148
	Reverse	5'-CCGACAGCACGAGGCTTTTT-3'	
Il2	Forward	5'-CTGCGGCATGTTCTGGATTT-3'	125
	Reverse	5'-TGGCACTCAAATGTGTTGTCAG-3'	
Il4	Forward	5'-CATCGGCATTTTGAACGAGGTCA-3'	240
	Reverse	5'-CTTATCGATGAATCCAGGCATCG-3'	
Il6	Forward	5'-CCACTTCACAAGTCGGAGGCTTA-3'	169
	Reverse	5'-CCAGTTTGGTAGCATCCATCATT-3'	
Il10	Forward	5'-AAGACAATAACTGCACCCACTTCC-3'	164
	Reverse	5'-GCAACCCAAGTAACCCTTAAAGTCC-3'	
Il17	Forward	5'-GGCCCTCAGACTACCTCAACC-3'	131
	Reverse	5'-CTTTCCCTCCGCATTGACAC-3'	
Mpst	Forward	5'-CATCAAGACCCACGAGGACA-3'	188
	Reverse	5'-TCTTCTCCAGGCCTTCGTTG-3'	
Tgfb	Forward	5'-ACATCACACGGGACCAAACC-3'	154
	Reverse	5'-AGGCACAGGGTCATCATCAAA-3'	
Tnfa	Forward	5'-GCCTCTTCTCATTCCTGCTTGT-3'	147
	Reverse	5'-ATGATCTGAGTGTGAGGGTCTGG-3'	

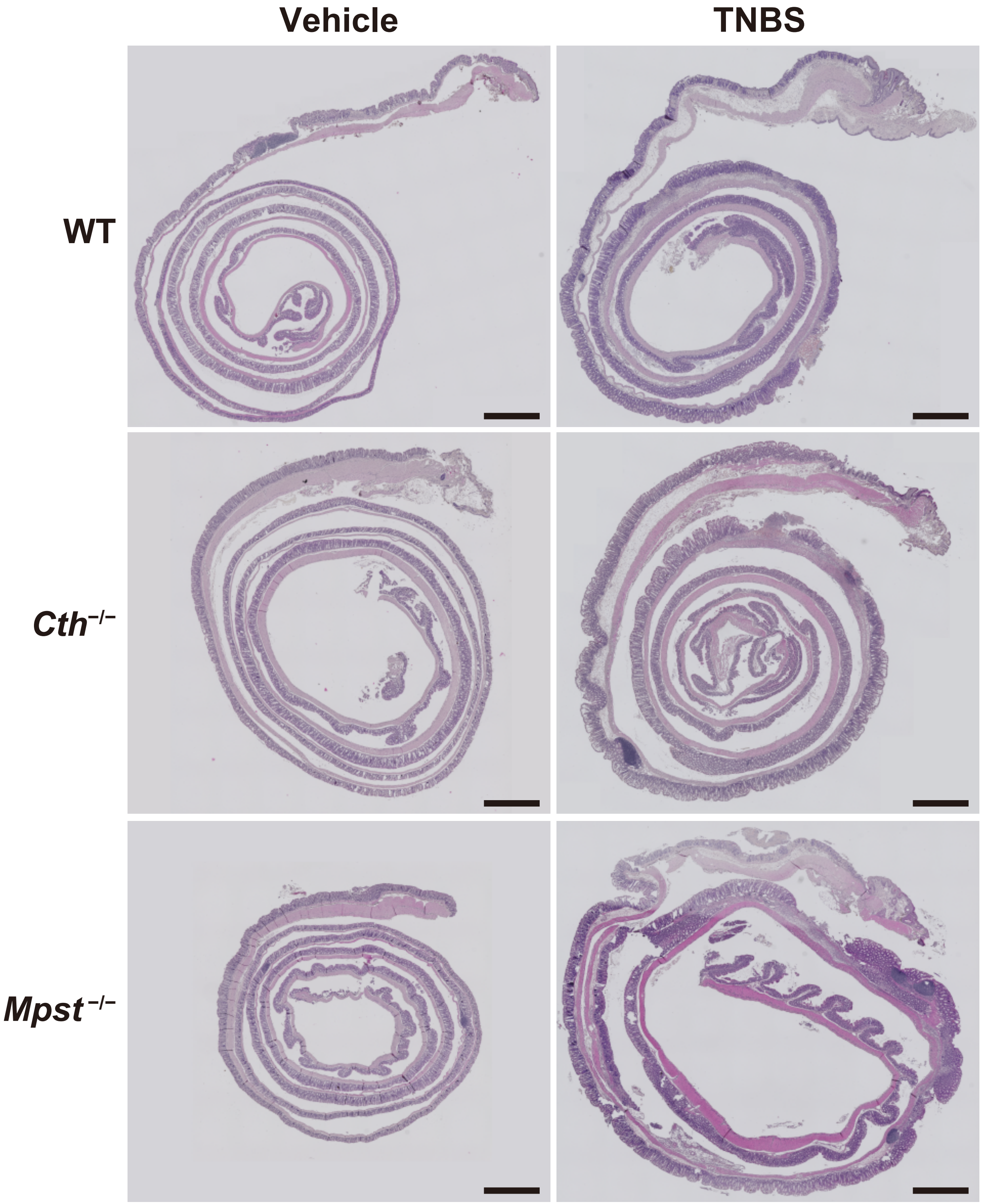

Supplementary Figure 1. Hematoxylin/eosin-stained colon sagittal sections of TNBS-induced colitis in wild-type (WT), CTH-deficient (*Cth*$^{-/-}$), and MPST-deficient (*Mpst*$^{-/-}$) mice. Bars indicate 1 mm.

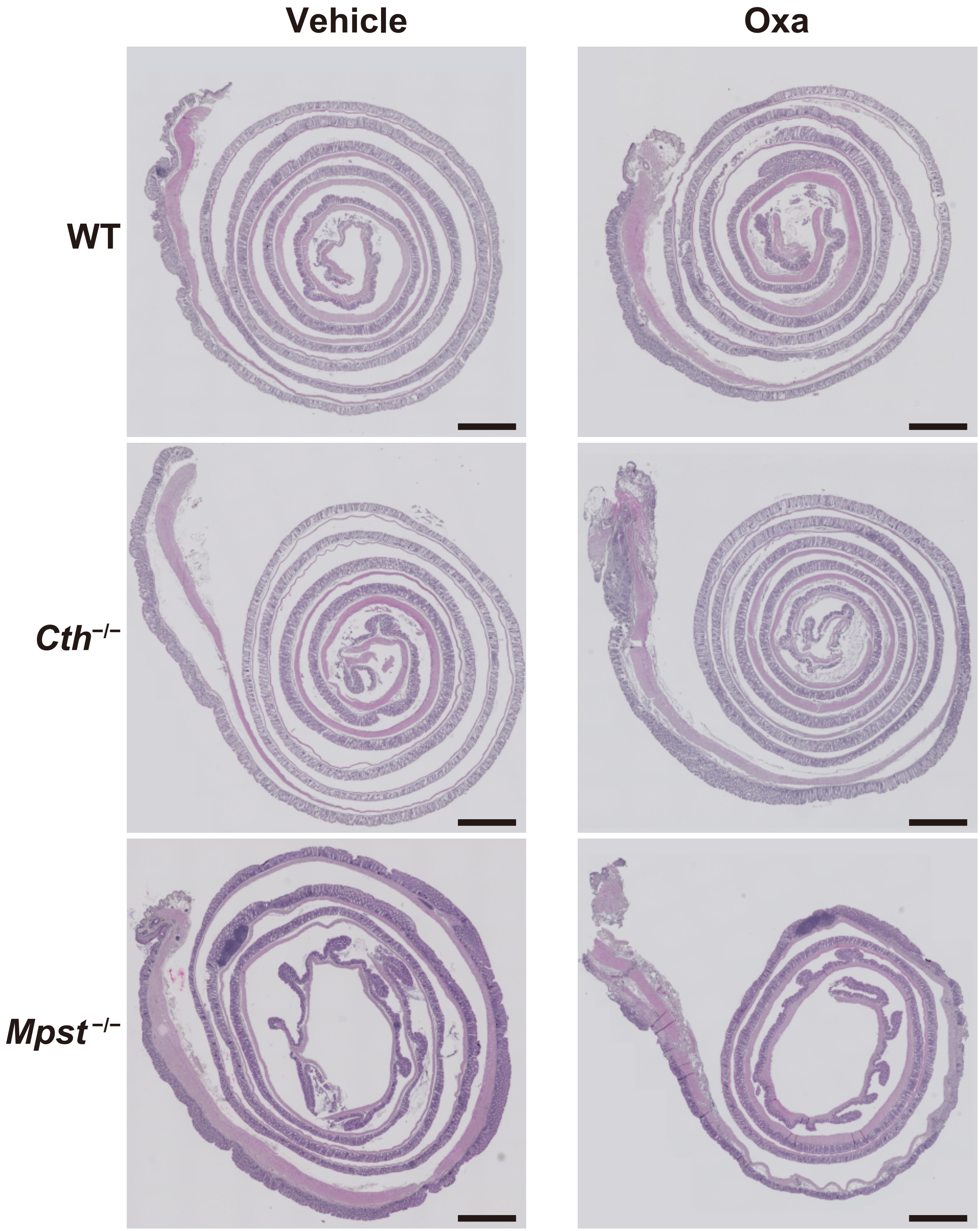

Supplementary Figure 2. Hematoxylin/eosin-stained colon sagittal sections of oxazolone-induced colitis in wild-type (WT), CTH-deficient (*Cth*$^{-/-}$), and MPST-deficient (*Mpst*$^{-/-}$) mice. Bars indicate 1 mm.

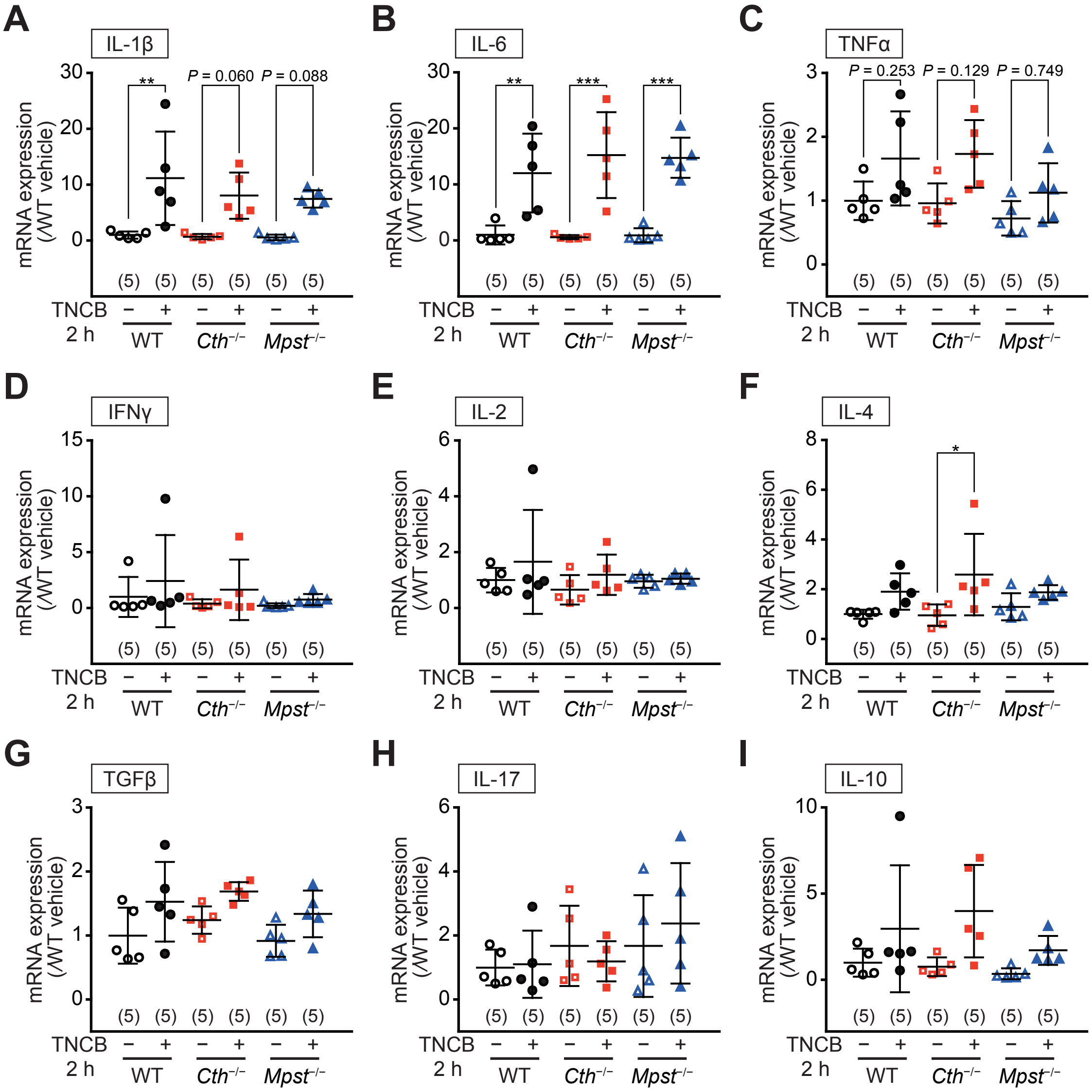

Supplementary Figure 3. Expression levels of various cytokine mRNAs in the ear during trinitrochlorobenzene (TNCB)-induced contact dermatitis at 2 h after the challenge. IL-1β (A), IL-6 (B), TNFα (C), IFNγ (D), IL-2 (E), IL-4 (F), TGFβ (G), IL-17 (H), and IL-10 (I) mRNA levels were normalized by the housekeeping HPRT1 mRNA levels and the relative expression *versus* vehicle-treated wild-type samples were calculated. Data are the mean ± SD with sample numbers in parentheses. Differences are significant by a one-way ANOVA with Tukey’ s multiple comparison test at **p*<0.05, ***p*<0.01, and ****p*<0.001 *versus* vehicle-treated samples of each genotype.
